# Prognostic implication of DPD quantification in transthyretin cardiac amyloidosis

**DOI:** 10.1093/ehjci/jeae295

**Published:** 2024-11-15

**Authors:** René Rettl, Franz Duca, Christina Kronberger, Christina Binder, Robin Willixhofer, Nikita Ermolaev, Michael Poledniczek, Felix Hofer, Christian Nitsche, Christian Hengstenberg, Roza Badr Eslam, Johannes Kastner, Jutta Bergler-Klein, Marcus Hacker, Raffaella Calabretta, Andreas A Kammerlander

**Affiliations:** Division of Cardiology, Department of Internal Medicine II, Medical University of Vienna, Vienna, Austria; Division of Cardiology, Department of Internal Medicine II, Medical University of Vienna, Vienna, Austria; Division of Cardiology, Department of Internal Medicine II, Medical University of Vienna, Vienna, Austria; Division of Cardiology, Department of Internal Medicine II, Medical University of Vienna, Vienna, Austria; Division of Cardiology, Department of Internal Medicine II, Medical University of Vienna, Vienna, Austria; Division of Cardiology, Department of Internal Medicine II, Medical University of Vienna, Vienna, Austria; Division of Cardiology, Department of Internal Medicine II, Medical University of Vienna, Vienna, Austria; Division of Cardiology, Department of Internal Medicine II, Medical University of Vienna, Vienna, Austria; Division of Cardiology, Department of Internal Medicine II, Medical University of Vienna, Vienna, Austria; Division of Cardiology, Department of Internal Medicine II, Medical University of Vienna, Vienna, Austria; Division of Cardiology, Department of Internal Medicine II, Medical University of Vienna, Vienna, Austria; Division of Cardiology, Department of Internal Medicine II, Medical University of Vienna, Vienna, Austria; Division of Cardiology, Department of Internal Medicine II, Medical University of Vienna, Vienna, Austria; Division of Nuclear Medicine, Department of Biomedical Imaging and Image-guided Therapy, Medical University of Vienna, Waehringer Guertel 18-20, 1090 Vienna, Austria; Division of Nuclear Medicine, Department of Biomedical Imaging and Image-guided Therapy, Medical University of Vienna, Waehringer Guertel 18-20, 1090 Vienna, Austria; Division of Cardiology, Department of Internal Medicine II, Medical University of Vienna, Vienna, Austria

**Keywords:** ATTR amyloidosis, ATTR-CA, DPD quantification, outcome, prognosis

## Abstract

**Aims:**

Quantification of cardiac [^99m^Tc]-3,3-diphosphono-1,2-propanodicarboxylic acid (DPD) uptake enhances diagnostic capabilities and may facilitate prognostic stratification in patients with transthyretin cardiac amyloidosis (ATTR-CA). This study aimed to evaluate the association of quantitative left ventricular (LV) DPD uptake with myocardial structure and function, and their implications on outcome in ATTR-CA.

**Methods and results:**

Consecutive ATTR-CA patients (*n* = 100) undergoing planar DPD scintigraphy with Perugini grade 2 or 3, alongside quantitative DPD single-photon emission computed tomography/computed tomography imaging and speckle-tracking echocardiography between 2019 and 2023, were included and divided into two cohorts based on median DPD retention index (low DPD uptake: ≤5.4, *n* = 50; high DPD uptake: >5.4, *n* = 50). The DPD retention index showed significant, albeit weak to modest, correlations with LV global longitudinal strain (LV-GLS: *r* = 0.366, *P* < 0.001), right ventricular free wall longitudinal strain (RV-FW-LS: *r* = 0.316, *P* = 0.002), LV diastolic function (E/e′ average: *r* = 0.304, *P* = 0.013), NT-proBNP (*r* = 0.332, *P* < 0.001), troponin T (*r* = 0.233, *P* = 0.022), 6 min walk distance (6MWD: *r* = −0.222, *P* = 0.033), and National Amyloidosis Centre (NAC) stage (*r* = 0.294, *P* = 0.003). ATTR-CA patients in the high DPD uptake cohort demonstrated more advanced disease severity regarding longitudinal cardiac function (LV-GLS: *P* = 0.012, RV-FW-LS: *P* = 0.036), LV diastolic function (E/e′ average: *P* = 0.035), cardiac biomarkers (NT-proBNP: *P* = 0.012, troponin T: *P* = 0.044), exercise capacity (6MWD: *P* = 0.035), and disease stage (NAC stage I: *P* = 0.045, III: *P* = 0.006), and experienced adverse outcomes compared with the low DPD uptake cohort [composite endpoint: all-cause death or heart failure hospitalization, HR: 2.873 (95% CI: 1.439–5.737), *P* = 0.003; DPD retention index: adjusted HR 1.221 (95% CI: 1.078–1.383), *P* = 0.002].

**Conclusion:**

In ATTR-CA, enhanced quantitative LV DPD uptake indicates advanced disease severity and is associated with adverse outcome. DPD quantification may facilitate prognostic stratification when diagnosing patients with ATTR-CA.

## Graphical Abstract

**Graphical Abstract:**
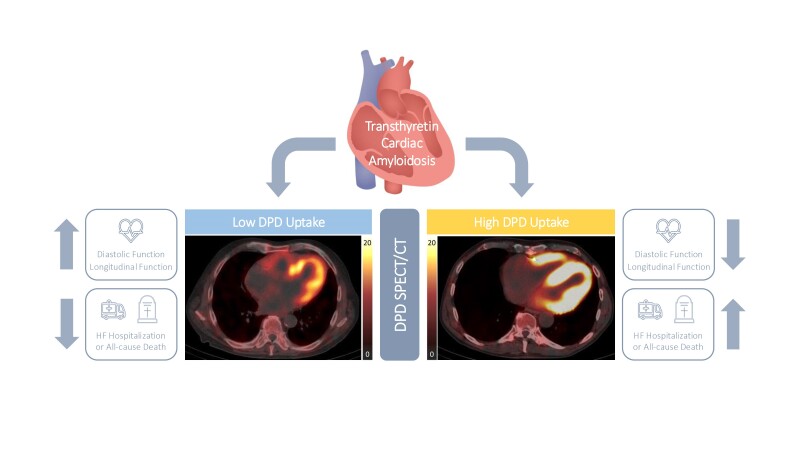
DPD, [^99m^Tc]-3,3-diphosphono-1,2-propanodicarboxylic acid; HF, heart failure; SPECT/CT, single-photon emission computed tomography/computed tomography.

## Introduction

Transthyretin cardiac amyloidosis (ATTR-CA) is delineated by the deposition of misfolded transthyretin (TTR) protein as insoluble amyloid fibrils within the myocardial extracellular space, culminating in restrictive heart failure (HF) with dismal prognosis, emphasizing the importance of early diagnosis.^1,2^ Non-invasive diagnostic approaches, including planar scintigraphy with bone-avid tracers such as [^99m^Tc]-3,3-diphosphono-1,2-propanodicarboxylic acid (DPD) or [^99m^Tc]-pyrophosphate (PYP), enables visualization of amyloid fibrils and have revolutionized the assessment and management of ATTR-CA.^3,4^ Quantitative single-photon emission computed tomography/computed tomography (SPECT/CT) imaging enhances diagnostic capabilities and may aid in facilitating prognostic stratification.^5^ Although planar DPD scintigraphy has been shown to have prognostic significance when comparing patients with normal and abnormal cardiac tracer uptake, it has not consistently revealed differences in outcomes among patients with confirmed cardiac amyloidosis (CA).^6,7^ Quantification of cardiac DPD uptake using SPECT/CT imaging potentially overcomes the limitations of planar DPD scintigraphy by providing three-dimensional visualization of left ventricular (LV) tracer uptake.^8–10^ However, the prognostic significance of quantitative LV DPD uptake remains poorly understood.

Therefore, the present study was designed to investigate the association of DPD quantification with myocardial structure and function, and their implications on outcome in patients with ATTR-CA.

## Methods

### Study design

The present study was conducted as part of a prospective patient registry at the Department of Cardiology, Medical University of Vienna, which encompasses a specialized outpatient clinic for CA. The study adhered to the principles of good clinical practice as outlined in the Declaration of Helsinki, was approved by the Ethics Committee of the Medical University of Vienna (EC #796/2010), and all participants provided written informed consent prior to enrolment.

### Study population

Consecutive registry patients diagnosed with ATTR-CA according to Perugini grade 2 or 3 between February 2019 and December 2023 were screened for study eligibility. ATTR-CA patients were excluded if the following criteria were met: (i) unavailability of, or inability to undergo, quantitative DPD SPECT/CT imaging; (ii) disease-specific therapy prior to quantitative DPD SPECT/CT imaging; and (iii) unavailability of, or inability to undergo, speckle-tracking echocardiography. Eligible study participants underwent baseline assessment at our specialized CA outpatient clinic as part of a prospective investigational imaging study.

### Diagnosis of transthyretin cardiac amyloidosis

Planar DPD scintigraphy was performed in patients with clinical and imaging evidence of CA, in accordance with current recommendations.^4^ ATTR-CA diagnosis relied on the presence of cardiac DPD uptake on planar scintigraphy (Perugini grade ≥ 2), confirmed by additional quantitative DPD SPECT/CT imaging, alongside the absence of paraprotein, or monoclonal protein detected by serum and urine immunofixation and serum free light chain assay.^4^ All study participants diagnosed with ATTR-CA were offered and accepted the option of TTR gene sequencing.

### DPD scintigraphy and SPECT/CT imaging

Nuclear imaging was performed at the Division of Nuclear Medicine, Department of Biomedical Imaging and Image-guided Therapy, Medical University of Vienna. A hybrid SPECT/CT system (Symbia Intevo, Siemens Medical Solutions AG, Erlangen, Germany) equipped with a low-energy, high-resolution collimator [dose length product: 85.0 mGy ∗ cm, interquartile range (IQR): 72.9–117.0] was used to obtain planar whole-body scintigraphy images 2.5 h and SPECT/CT images of the thorax 3.0 h after intravenous injection of DPD (activity: 720.0 MBq, IQR: 707.0–739.0). Images were captured in a 180° configuration, comprising 64 views with a duration of 20 s per view, a 256 × 256 matrix, and an energy window of 15% around the DPD photopeak of 142 keV. After the SPECT acquisition, a low-dose CT scan was obtained for attenuation correction, using settings of 130 kV, 35 mAs, a 256 × 256 matrix, and a step-and-shoot acquisition with body contour. Image acquisition and reconstruction were performed using xSPECT/CT QUANT (xQUANT) technology (eight iterations, four subsets, 3.0 mm smoothing filter, and a 20 mm Gaussian filter), which uses a 3% National Institute of Standards and Technology traceable precision source to standardize uptake values across different cameras, dose calibrators, and equipment.^11,12^ Nuclear medicine images were independently reviewed by two experienced nuclear medicine physicians. Planar DPD scintigraphy images were graded visually according to Perugini *et al*.^3^ while LV DPD uptake on SPECT/CT images was quantified using dedicated software (Hermes Hybrid 3D software, Hermes Medical Solutions, Stockholm, Sweden).

### DPD quantification

**Figure 1:**
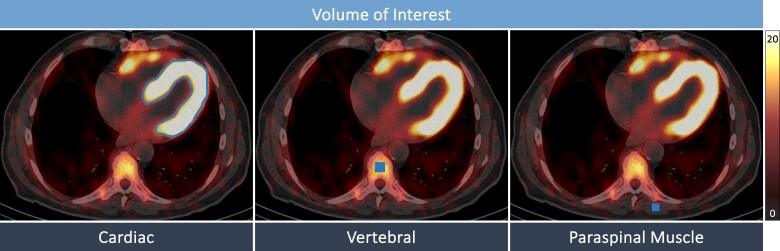
DPD quantification. DPD SPECT/CT images of a representative ATTR-CA patient demonstrating the VOI for each tissue (VOI cardiac: automatically generated using a threshold-based method, VOI vertebral: 2.92 mL, VOI paraspinal muscle: 1.19 mL) placed to quantify the SUV. ATTR-CA, transthyretin cardiac amyloidosis; DPD, [^99m^Tc]-3,3-diphosphono-1,2-propanodicarboxylic acid; SPECT/CT, single-photon emission computed tomography/computed tomography; SUV, standardized uptake value; VOI, volume of interest.

Three-dimensional volumes of interest (VOIs) for the LV myocardium were automatically generated by dedicated software using a threshold-based method (39% of maximal activity, as previously described,^8–10^) allowing clear separation of LV wall from LV cavity blood pool activity (*Figure 1*), and were operator-adjusted for apparent sternal or rib uptake. From these VOI, the standardized uptake value (SUV) was calculated, indicating the concentration of the radiopharmaceutical in the respective tissue, with the SUV peak representing the highest average SUV within a 1 cm^3^ volume.^8–10^ Bone uptake (SUV peak vertebral) was calculated by manual placement of a cubic VOI (2.92 mL) in an intact vertebral body of a thoracic spine in an area without degenerative changes to minimize bias from high degenerative tracer accumulation.^8–10^ Soft tissue uptake (SUV peak paraspinal muscle) was determined by manually placing a cubic VOI (1.19 mL) in the left paraspinal muscle.^8–10^ Confounding arising from competing tracer uptake among tissues was corrected by calculating a composite DPD retention index according to the following formula:


$$\begin{array}{rl} \mathrm{DPD}\;\mathrm{retention}\;\mathrm{index}\;= & \left(\frac{\mathrm{SUV}\;\mathrm{peak}\;\mathrm{cardiac}}{\mathrm{SUV}\;\mathrm{peak}\;\mathrm{vertebral}}\right)\\ & \times \;\mathrm{SUV}\;\mathrm{peak}\;\mathrm{paraspinal}\;\mathrm{muscle} \end{array}.$$

### Echocardiographic imaging

Transthoracic echocardiography was performed by board-certified and experienced operators on high-end equipment (GE Vivid E95 and Vivid 7, GE Healthcare, Wauwatosa, WI, USA) according to current recommendations, including two-dimensional speckle-tracking analysis of the LV and right ventricle (RV).^13^ LV global longitudinal strain (GLS) was measured by tracking the myocardium in apical two-, three-, and four-chamber views.^14^ The RV free wall longitudinal strain (RV-FW-LS) was measured in the free lateral RV wall in an optimized apical four-chamber view.^15^ LV filling pressure and diastolic function were estimated by dividing the mitral inflow E-wave velocity by the early diastolic mitral annular velocity (E/e′).^16^ Image analyses were performed after image acquisition on a modern offline clinical workstation equipped with dedicated software (EchoPAC, GE Healthcare, Wauwatosa, WI, USA) by board-certified cardiologists.

### Outcome measures

The outcome endpoint was a composite of all-cause death or HF hospitalization. HF-related hospitalizations were defined as events associated with worsening of dyspnoea, and/or weight gain, and/or peripheral oedema requiring hospital admission and/or intravenous diuretic therapy. Outcome events were recorded during follow-up at our outpatient clinic or via telephone visits, and were additionally obtained from electronic medical records, along with the Austrian death registry.

### Statistical analysis

All statistical analyses were performed using SPSS version 29 (IBM Corp, New York, NY, USA). Continuous variables are expressed as median and IQR, and categoric variables as numbers and percentages. The Mann–Whitney *U* test or χ^2^ test was used to compare between two cohorts, and Spearman’s correlation (*r*) was used to express correlations. Kaplan–Meier plots and corresponding log-rank tests were used to verify the time-dependent discriminative power of the DPD retention index on the composite outcome endpoint. Univariable Cox regression models were calculated to assess the effect of imaging, laboratory and clinical variables on the composite outcome endpoint. Variables that significantly predicted outcome were subsequently entered into a stepwise forward multivariable Cox regression model to adjust for potential confounding effects. A *P*-value of ≤0.05 was considered statistically significant.

## Results

### Study participants

**Figure 2:**
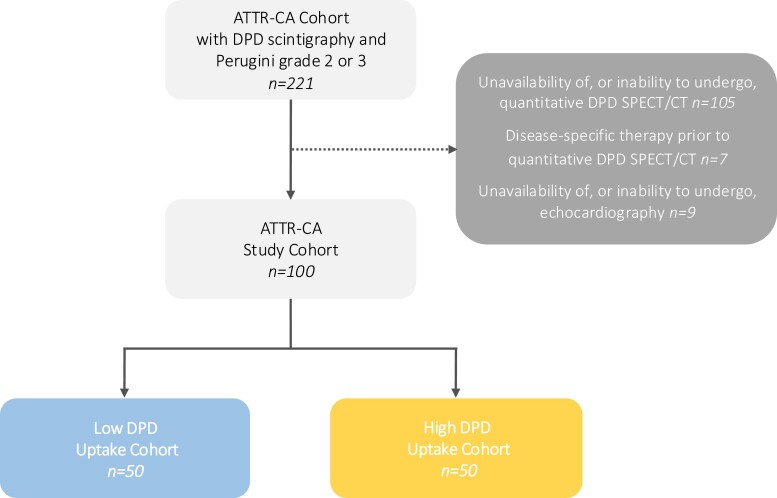
Patient flowchart. A total of 221 ATTR-CA patients who underwent planar DPD scintigraphy demonstrating Perugini grade 2 or 3 were screened for study eligibility. Reasons for study exclusion are presented. ATTR-CA, transthyretin cardiac amyloidosis; DPD, [^99m^Tc]-3,3-diphosphono-1,2-propanodicarboxylic acid; SPECT/CT, single-photon emission computed tomography/computed tomography.

Among 221 ATTR-CA patients who underwent planar DPD scintigraphy demonstrating Perugini grade 2 or 3 between February 2019 and December 2023, 100 individuals undergoing quantitative DPD SPECT/CT imaging and speckle-tracking echocardiography were considered eligible; specific reasons for study exclusion or discontinuation are depicted in *Figure 2*. ATTR-CA patients were classified into two cohorts based on median DPD retention index (low DPD uptake: ≤5.4, *n* = 50; high DPD uptake: >5.4, *n* = 50).

### Baseline characteristics

Detailed baseline characteristics for the entire study cohort are depicted in *Table 1*. ATTR-CA patients were elderly (79.0 years, IQR: 75.0–84.0), predominantly male (79.0%), and gene sequencing identified thirteen patients (13.0%) with a mutation in the TTR gene (hereditary variant ATTR). Nearly half of ATTR-CA patients were in an advanced stage of HF (New York Heart Association (NYHA) functional class ≥ III: 46.0%) and exercise capacity assessed by 6 min walk distance was impaired (6MWD: 363.0 m, IQR: 260.5–463.0). Cardiac biomarker levels including N-terminal prohormone of brain natriuretic peptide (NT-proBNP: 2341 ng/L, IQR: 1248–3921) and troponin T (52.0 ng/L, IQR: 32.0–72.0) were markedly elevated. The National Amyloidosis Centre (NAC) staging system classified 52.0% of ATTR-CA patients into NAC stage I, 32.0% into NAC stage II, and 16.0% into NAC stage III. Nuclear imaging with planar DPD scintigraphy graded 28.0% of ATTR-CA patients as Perugini 2 and 72.0% as Perugini 3, while quantitative DPD SPECT/CT imaging demonstrated increased LV tracer uptake (DPD retention index: 5.4, IQR: 3.4–7.9). Echocardiographic assessment unveiled a prominent interventricular septum (IVS: 19.0 mm, IQR: 16.0–22.0), a mildly reduced LV ejection fraction (LVEF: 49.0%, IQR: 40.0–55.0), significantly impaired longitudinal cardiac function as reflected by LV-GLS (11.0%, IQR: 14.1–8.7) and RV-FW-LS (14.1%, IQR: 17.3–9.0), and marked LV diastolic dysfunction (E/e′ average: 17.8, IQR: 14.7–23.4).

**Table 1:** Baseline characteristics and cohort comparison according to DPD retention index

	All patients (*n* = 100)	Low DPD uptake (*n* = 50)	High DPD uptake (*n* = 50)	*P*
Demographics				
Age, years	79.0 (75.0–84.0)	79.0 (75.0–82.0)	80.0 (75.0–86.0)	0.102
Male sex	79 (79.0)	43 (86.0)	36 (72.0)	0.086
Body mass index, kg/m²	25.7 (22.6–28.4)	24.5 (22.5–27.7)	26.2 (23.9–29.4)	0.075
ATTR variant	13 (13.0)	7 (14.0)	6 (12.0)	0.766
Comorbidities				
Atrial fibrillation/flutter	62 (62.0)	28 (56.0)	34 (68.0)	0.216
Cardiac device	23 (23.0)	11 (22.0)	12 (24.0)	0.812
Polyneuropathy	61 (61.0)	28 (56.0)	33 (66.0)	0.305
Carpal tunnel syndrome	48 (48.0)	23 (46.0)	25 (50.0)	0.689
Medications				
Beta-blockers	45 (45.0)	23 (46.0)	22 (44.0)	0.841
Diuretic agent	76 (76.0)	35 (70.0)	41 (82.0)	0.160
Mineralocorticoid receptor antagonist	53 (53.0)	24 (48.0)	29 (58.0)	0.316
Clinical characteristics				
NYHA functional class ≥ III	46 (46.0)	18 (36.0)	28 (56.0)	**0**.**045**
NAC stage				
I	52 (52.0)	31 (62.0)	21 (42.0)	**0**.**045**
II	32 (32.0)	16 (32.0)	16 (32.0)	1.000
III	16 (16.0)	3 (6.0)	13 (26.0)	**0**.**006**
6** **min walk distance, m	363.0 (260.5–463.0)	400.0 (324.0–475.0)	346.5 (230.0–445.0)	**0**.**035**
Laboratory characteristics				
NT-proBNP, ng/L	2341 (1248–3921)	1734 (989–3254)	2922 (1909–4324)	**0**.**012**
Troponin T, ng/L	52.0 (32.0–72.0)	51.5 (31.0–69.5)	55.0 (35.0–101.0)	**0**.**044**
eGFR, mL/min/1.73** **m^2^	59.3 (44.0–78.7)	62.5 (49.0–79.9)	55.5 (40.0–74.1)	0.106
Nuclear imaging characteristics				
Perugini grade 2	28 (28.0)	16 (32.0)	12 (24.0)	0.373
Perugini grade 3	72 (72.0)	34 (68.0)	38 (76.0)	0.373
DPD retention index	5.4 (3.4–7.9)	3.4 (2.1–4.0)	7.9 (6.6–10.8)	**<0**.**001**
DPD activity, MBq	720.0 (707.0–739.0)	727.0 (710.0–742.0)	715.0 (707.0–731.0)	0.138
DLP, mGy ∗ cm	85.0 (72.9–117.0)	85.5 (72.0–115.0)	85.0 (73.5–118.0)	0.660
Echocardiographic characteristics				
Interventricular septum, mm	19.0 (16.0–22.0)	19.0 (16.0–22.0)	19.0 (17.0–21.0)	0.974
LV end-diastolic diameter, mm	41.5 (37.0–46.0)	42.0 (39.0–46.0)	41.0 (37.0–44.0)	0.323
LV ejection fraction, %	49.0 (40.0–55.0)	50.0 (43.6–55.0)	46.0 (40.0–54.8)	0.471
LV global longitudinal strain, −%	11.0 (14.1–8.7)	12.5 (14.7–9.9)	10.1 (12.4–8.2)	**0**.**012**
E/e**′** septal^a^	20.8 (16.8–25.5)	19.0 (16.5–22.4)	24.2 (17.0–30.3)	**0**.**038**
E/e**′** lateral^a^	15.3 (12.4–20.2)	13.8 (12.3–17.8)	17.2 (12.4–22.8)	**0**.**042**
E/e**′** average^a^	17.8 (14.7–23.4)	16.6 (14.7–20.3)	21.2 (15.2–26.5)	**0**.**035**
RV end-diastolic diameter, mm	33.0 (30.0–37.0)	33.0 (29.0–36.0)	33.0 (30.0–37.0)	0.734
RV free wall longitudinal strain, −%	14.1 (17.3–9.0)	15.0 (19.3–10.0)	11.7 (17.0–9.0)	**0**.**036**
LA volume index, mL/m^2^	35.1 (29.1–48.3)	34.2 (26.3–49.4)	36.0 (31.1–47.7)	0.618
RA volume index, mL/m^2^	32.8 (25.2–40.1)	32.8 (24.6–40.6)	32.9 (25.9–39.8)	0.803
TR velocity, m/s	3.0 (2.6–3.3)	2.9 (2.6–3.2)	3.0 (2.7–3.3)	0.213
sPAP, mmHg	48.0 (41.0–53.0)	48.0 (39.0–51.0)	48.0 (41.0–54.0)	0.586

Continuous variables are expressed as median and interquartile range and categoric variables as numbers and percentages. Bold indicates *P* ≤ 0.05.

ATTR, transthyretin amyloid; DLP, dose length product; DPD, [^99m^Tc]-3,3-diphosphono-1,2-propanodicarboxylic acid; E/e′, mitral inflow E-wave/early diastolic mitral annular velocity; eGFR, estimated glomerular filtration rate; LA, left atrium; LV, left ventricle; NAC, National Amyloidosis Centre; NT-proBNP, N-terminal prohormone of brain natriuretic peptide; NYHA, New York Heart Association; RA, right atrium; RV, right ventricle; sPAP, systolic pulmonary artery pressure; TR, tricuspid regurgitation.

^a^E/e′ available for *n* = 66 (low DPD uptake: *n* = 35, high DPD uptake: *n* = 31).

### Cohort comparison of baseline characteristics

Differences in baseline characteristics between cohorts are presented in *Table 1*. Comparison of nuclear imaging parameters (low DPD uptake, *n* = 50 vs. high DPD uptake, *n* = 50) revealed significant differences in the DPD retention index (3.4 vs. 7.9, *P* < 0.001), although no differences according to Perugini classification (grade 2: 32.0% vs. 24.0%, grade 3: 68.0% vs. 76.0%, *P* = 0.373). When comparing echocardiographic parameters, significant differences in longitudinal cardiac function (LV-GLS: −12.5% vs. −10.1%, *P* = 0.012; RV-FW-LS: −15.0% vs. −11.7%, *P* = 0.036) and LV diastolic function (E/e′ average: 16.6 vs. 21.2, *P* = 0.035) became apparent. Juxtaposition of cardiac biomarkers yielded notable differences in serum levels of NT-proBNP (1734.0 ng/L vs. 2922.0 ng/L, *P* = 0.012) and troponin T (51.5 ng/L vs. 55.0 ng/L, *P* = 0.044), and comparison of exercise capacity showed significant differences in 6MWD (400.0 m vs. 346.5 m, *P* = 0.035). Regarding disease stage, noteworthy disparities in NAC stage were observed between the cohorts (NAC I: 62.0% vs. 42.0%, *P* = 0.045; NAC III: 6.0% vs. 26.0%, *P* = 0.006). Cohort comparison according to the Perugini classification is shown in Supplementary data online, *Table S1*.

### Correlation of DPD quantification with echocardiographic, laboratory, and clinical characteristics

**Figure 3:**
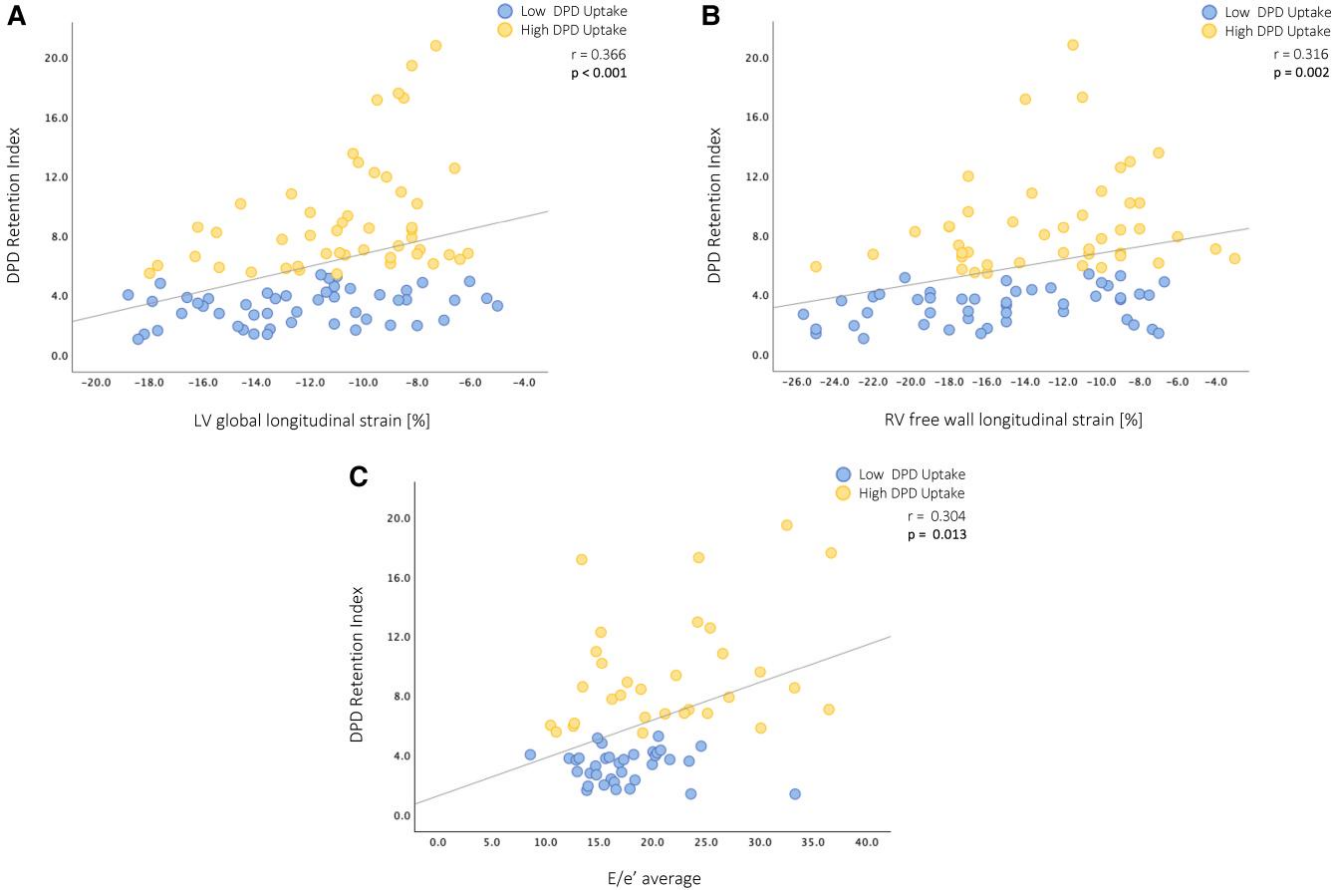
Correlation: DPD quantification and echocardiographic characteristics. (*A*) Correlation between DPD retention index and LV global longitudinal strain. (*B*) Correlation between DPD retention index and RV free wall longitudinal strain. (*C*) Correlation between DPD retention index and LV diastolic function. DPD, [^99m^Tc]-3,3-diphosphono-1,2-propanodicarboxylic acid; E/e′, mitral inflow E-wave/early diastolic mitral annular velocity; LV, left ventricle; RV, right ventricle.

**Figure 4:**
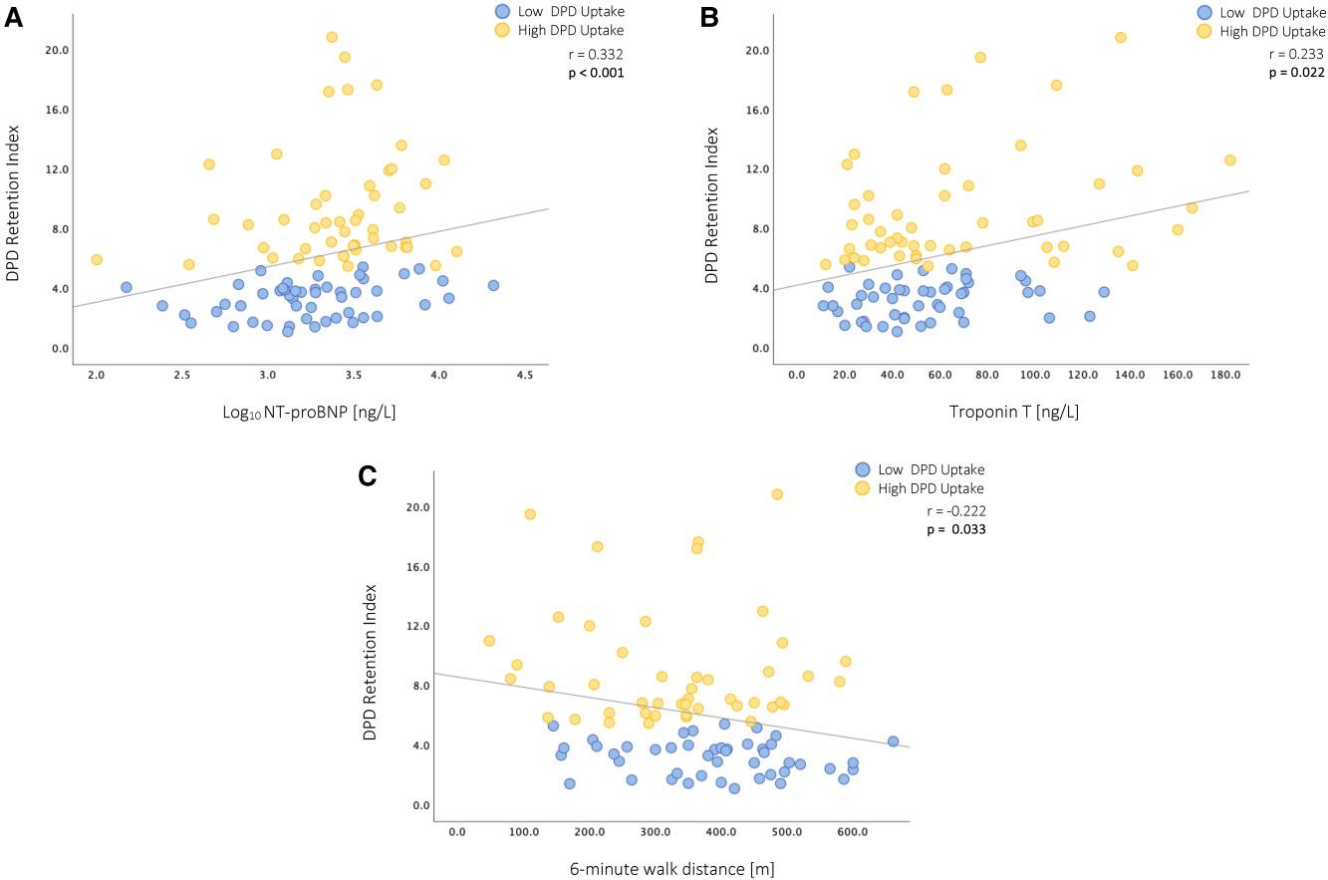
Correlation: DPD quantification and clinical and laboratory characteristics. (*A*) Correlation between DPD retention index and NT-proBNP. (*B*) Correlation between DPD retention index and troponin T. (*C*) Correlation between DPD retention index and 6 min walk distance. DPD, [^99m^Tc]-3,3-diphosphono-1,2-propanodicarboxylic acid; NT-proBNP, N-terminal prohormone of brain natriuretic peptide.

Detailed correlation analyses are depicted in *Table 2*. Comparison of quantitative LV DPD uptake with echocardiographic, laboratory, and clinical parameters in ATTR-CA patients revealed significant, albeit weak to modest, correlations between the DPD retention index and longitudinal cardiac function (LV-GLS: *r* = 0.366, *P* < 0.001, *Figure 3A*; RV-FW-LS: *r* = 0.316, *P* = 0.002, *Figure 3B*), LV diastolic function (E/e′ average: *r* = 0.304, *P* = 0.013, *Figure 3C*), cardiac biomarkers (NT-proBNP: *r* = 0.332, *P* < 0.001, *Figure 4A*; troponin T: *r* = 0.233, *P* = 0.022, *Figure 4B*), exercise capacity (6MWD: *r* = −0.222, *P* = 0.033, *Figure 4C*), and disease stage (NAC stage I-III: *r* = 0.294, *P* = 0.003).

**Table 2:** Correlation analysis of DPD retention index with clinical, laboratory, and imaging characteristics

All patients (*n* = 100)	Correlation coefficient (*r*)	*P*
NYHA functional class ≥ III	0.198	**0**.**048**
NAC stage	0.294	**0**.**003**
6** **min walk distance	−0.222	**0**.**033**
Log_10_ NT-proBNP	0.332	**<0**.**001**
Troponin T	0.233	**0**.**022**
Perugini grade	0.173	0.086
Interventricular septum	0.130	0.203
LV end-diastolic diameter	−0.185	0.068
LV ejection fraction	−0.058	0.571
LV global longitudinal strain	0.366	**<0**.**001**
E/e**′** average^a^	0.304	**0**.**013**
RV end-diastolic diameter	−0.016	0.875
RV free wall longitudinal strain	0.316	**0**.**002**
LA volume index	0.025	0.812
RA volume index	0.068	0.510
TR velocity	0.134	0.202
sPAP	0.153	0.176

*r* indicates Spearman’s correlation coefficient. Bold indicates *P* ≤ 0.05. Abbreviations as in *Table 1*.

^a^E/e′ available for *n* = 66.

### Impact of DPD quantification on outcome

During a median follow-up period of 21.0 months (IQR: 6.0–38.5), which did not differ between cohorts (*P* = 0.712), 41 (41.0%) ATTR-CA patients reached the composite endpoint of all-cause death (*n* = 20, 20.0%) or HF hospitalization (*n* = 21, 21.0%). In the low DPD uptake cohort, the composite endpoint occurred in 11 (22.0%) patients (all-cause death: *n* = 5, 10.0%; HF hospitalization: *n* = 6, 12.0%), whereas in the high DPD uptake cohort, 30 (60.0%) patients had an outcome event (all-cause death: *n* = 15, 30.0%; HF hospitalization: *n* = 15, 30.0%).

**Figure 5:**
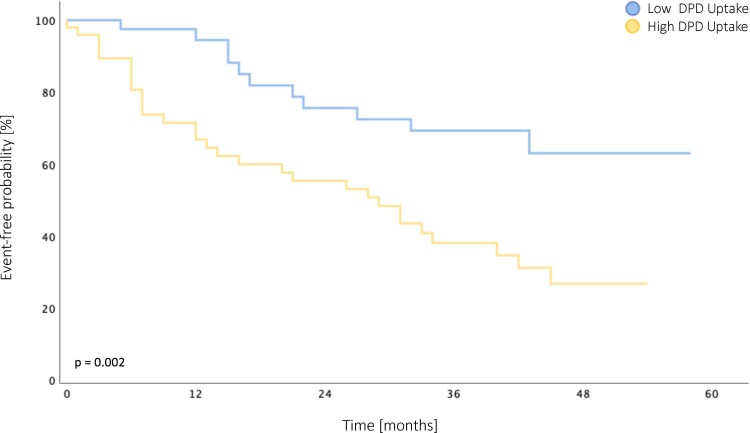
Kaplan–Meier plot for the composite endpoint of all-cause death or heart failure hospitalization stratified by DPD retention index.

Kaplan–Meier analysis for the composite endpoint of all-cause death or HF hospitalization showed that ATTR-CA patients with a quantitative LV DPD uptake above the median had a significantly shorter event-free survival [*P* = 0.002, *Figure 5*; hazard ratio (HR): 2.873 (95% confidence interval (CI): 1.439–5.737), *P* = 0.003]. In contrast, stratification according to the Perugini classification demonstrated no difference in outcome between Perugini grades 2 and 3 [*P* = 0.126, Supplementary data online, *Figure S1*; HR: 1.861 (95% CI: 0.825–4.202), *P* = 0.135].

Univariable Cox regression models for the composite endpoint of all-cause death or HF hospitalization (*Table 3*) revealed that the DPD retention index predicted adverse outcome [HR: 1.123 (95% CI: 1.059–1.190), *P* < 0.001], alongside with NYHA functional class [HR: 1.962 (95% CI: 1.028–3.745), *P* = 0.041], NAC stage [HR: 2.417 (95% CI: 1.656–3.529), *P* < 0.001], 6MWD [HR: 0.996 (95% CI: 0.994–0.999), *P* = 0.002], NT-proBNP [HR: 4.388 (95% CI: 1.917–10.044), *P* < 0.001], troponin T [HR: 1.015 (95% CI: 1.008–1.022), *P* < 0.001], LV-GLS [HR: 1.164 (95% CI: 1.051–1.290), *P* = 0.004], and RV-FW-LS [HR: 1.109 (95% CI: 1.040–1.183), *P* = 0.002]. After adjustment in the multivariable model, only the DPD retention index [HR: 1.221 (95% CI: 1.078–1.383), *P* = 0.002] and the NAC stage [HR: 5.301 (95% CI: 2.245–12.517), *P* < 0.001] remained predictive.

**Table 3:** Cox regression analyses for the composite endpoint of all-cause death or heart failure hospitalization

All patients (*n* = 100)	Univariable HR (95% CI)	*P*	Multivariable HR (95% CI)	*P*
Age	1.012 (0.975–1.051)	0.518		
Sex	0.735 (0.375–1.444)	0.372		
Body mass index	1.004 (0.944–1.067)	0.898		
NYHA functional class ≥ III	1.962 (1.028–3.745)	**0**.**041**	1.695 (0.666–4.313)	0.268
NAC stage	2.417 (1.656–3.529)	**<0**.**001**	5.301 (2.245–12.517)	**<0**.**001**
6** **min walk distance	0.996 (0.994–0.999)	**0**.**002**	0.997 (0.993–1.001)	0.142
Log_10_ NT-proBNP	4.388 (1.917–10.044)	**<0**.**001**	0.541 (0.074–3.933)	0.543
Troponin T	1.015 (1.008–1.022)	**<0**.**001**	0.994 (0.981–1.007)	0.378
Perugini grade	1.861 (0.825–4.202)	0.135		
DPD retention index	1.123 (1.059–1.190)	**<0**.**001**	1.221 (1.078–1.383)	**0**.**002**
Interventricular septum	1.003 (0.926–1.086)	0.936		
LV end-diastolic diameter	0.990 (0.947–1.035)	0.662		
LV ejection fraction	0.978 (0.949–1.008)	0.151		
LV global longitudinal strain	1.164 (1.051–1.290)	**0**.**004**	0.928 (0.784–1.098)	0.381
E/e**′** average^a^	1.055 (0.999–1.114)	0.053		
RV end-diastolic diameter	1.008 (0.955–1.064)	0.776		
RV free wall longitudinal strain	1.109 (1.040–1.183)	**0**.**002**	1.090 (0.981–1.211)	0.111
LA volume index	0.996 (0.974–1.018)	0.726		
RA volume index	1.000 (0.976–1.024)	0.995		
TR velocity	1.624 (0.893–2.955)	0.112		
sPAP	1.008 (0.980–1.037)	0.571		

Bold indicates *P* ≤ 0.05. Abbreviations as in *Table 1*.

HR, hazard ratio; CI, confidence interval.

^a^E/e′ available for *n* = 66.

## Discussion

Quantification of cardiac DPD uptake enhances diagnostic capabilities and may facilitate prognostic stratification in patients with ATTR-CA.^8–17^ In the present study, we systematically investigated the association of DPD quantification using SPECT/CT imaging with echocardiographic and clinical characteristics, and their implication on outcome in ATTR-CA. We demonstrated that: (i) quantitative LV DPD uptake showed significant, albeit weak to modest, correlations with longitudinal and diastolic cardiac function, cardiac biomarkers, exercise capacity, and disease stage; (ii) enhanced quantitative LV DPD uptake is indicative of more advanced disease severity, and (iii) is associated with adverse outcome. Therefore, DPD quantification may provide a valuable tool for prognostic stratification in patients diagnosed with ATTR-CA.

In the present study, 100 ATTR-CA patients underwent quantitative DPD SPECT/CT imaging and speckle-tracking echocardiography. We observed significant differences between the low and high DPD uptake cohorts with respect to LV-GLS and RV-FW-LS, indicating that a higher DPD retention index is accompanied with more severe LV and RV longitudinal dysfunction. In contrast, classification according to Perugini grading revealed no differences in longitudinal cardiac function between Perugini grades 2 and 3. Our results are consistent with recent findings demonstrating that increasing quantitative cardiac DPD uptake correlates with more pronounced impairment of both LV and RV longitudinal function in patients diagnosed with ATTR-CA.^18,19^ Pathophysiologically, the accumulation of amyloid fibrils in the myocardium impairs cardiac function,^1,2^ initially affecting longitudinal cardiac function and, in later stages of the disease, radial cardiac function, leading to more severe systolic dysfunction.^20,21^ Considering the predominance of NAC stage I ATTR-CA patients in our study population, we did not notice notable differences in LVEF between the low and high DPD uptake cohorts. Conversely, pronounced diastolic dysfunction, resulting from increased ventricular stiffness and elevated LV filling pressures, is pathognomonic for CA and can be observed even in the early stages of the disease.^22^ This characteristic pattern of early diastolic impairment is highlighted in the present study, as significant differences in diastolic function were observed between ATTR-CA patients in the low and high DPD uptake cohorts, suggesting that enhanced quantitative LV DPD uptake is associated with more severe diastolic dysfunction. In contrast, ATTR-CA patients with Perugini grade 2 showed no significant differences in diastolic function compared with those with Perugini grade 3.

Alongside impaired cardiac function, individuals with CA suffer from symptoms of HF and declined functional capacity, particularly in advanced stages of the disease.^1,2^ In our current investigation, we observed significant differences in NT-proBNP, troponin T, and 6MWD between ATTR-CA patients in the low and high DPD uptake cohorts, implying that a higher DPD retention index is related to increased cardiac biomarkers, diminished exercise capacity, and advanced disease severity. These results are in line with previous studies reporting a correlation between quantitative cardiac DPD uptake and NT-proBNP as well as troponin T levels.^18,19^ In contrast, data from a planar PYP imaging study correlating semi-quantitative metrics such as heart-to-contralateral lung (H/CL) ratio with cardiac biomarkers showed a strong association with troponin T but not NT-proBNP, highlighting the superiority of quantitative over semi-quantitative imaging approaches.^8,23^

The progressive nature of the disease is associated with a dismal prognosis, which can be assessed using a staging system that classifies ATTR-CA patients into prognostic categories, with a higher NAC stage indicating a less favourable outcome.^24^ In the present study, we noted a positive correlation between quantitative LV DPD uptake and NAC stage, suggesting that the DPD retention index may have prognostic implication in ATTR-CA. Despite the high diagnostic sensitivity of planar DPD scintigraphy in ATTR-CA, its prognostic value remains limited, as results from a large planar DPD imaging study failed to show differences in prognosis between patients with distinct grades of abnormal cardiac uptake (Perugini grade 1, 2, or 3).^6^ Although recent evidence highlighting prognostic significance when comparing patients with low-grade (Perugini grade 1) and high-grade (Perugini grade ≥ 2) cardiac DPD uptake, it has not consistently revealed outcome differences in patients with confirmed ATTR-CA when comparing Perugini grade 2 and grade 3.^7^ These findings are in line with our present study and suggest that the specific grade of positivity according to the Perugini classification provides limited prognostic information. In contrast, data from a multicentre planar PYP scintigraphy study utilizing semi-quantitative measures of myocardial retention using the H/CL ratio reported an independent association with survival in ATTR-CA patients.^25^ This raises the intriguing possibility that semi-quantitative or quantitative measures may provide prognostic insights beyond those provided by the Perugini classification, which can be confounded by soft tissue tracer uptake.

Utilizing quantitative measurement of LV DPD uptake with SPECT/CT imaging, we were able to demonstrate that ATTR-CA patients with a high DPD retention index face a more adverse outcome than those with less quantitative LV DPD uptake, emphasizing the sensitivity of quantitative over planar imaging approaches.^8^ Further evidence of the prognostic value of quantitative imaging in ATTR-CA was provided by Miller *et al*.,^26^ utilizing PYP SPECT quantification to assess the volume of involvement and cardiac PYP activity (CPA), which were associated with the incidence of HF hospitalizations. In addition, the present study observed significant, albeit weak to modest, correlations between the DPD retention index and both LV and RV longitudinal function, as well as cardiac biomarkers including NT-proBNP and troponin T, along with exercise capacity. These markers collectively reflect disease severity in ATTR-CA, thereby reinforcing the value of quantitative LV DPD uptake as a predictive outcome marker.^24,27,28^ This is consistent with the results of a quantitative PYP SPECT/CT imaging study, where volumetric parameters including CPA and cardiac PYP volume (CPV) were found to correlate with disease burden and prognostic factors in ATTR-CA.^29^ In addition, recent evidence has shown an association of cardiac DPD uptake with histological amyloid burden,^30^ facilitating the application of DPD quantification for prognostic stratification when diagnosing patients with ATTR-CA.

### Limitations

The present study harbours specific limitations that merit discussion. First, given the single-centre design of our study, centre-specific and selection bias cannot be completely excluded. However, confining data collection to a single centre provides considerable advantages in maintaining consistency across the diagnostic work-up and baseline assessments. Secondly, the sample size is constrained due to the single-centre design and the rarity of ATTR-CA, and patients with Perugini grade 0 or 1, representing a rare subset in CA, were not included. However, this study marks the pioneering effort to investigate the prognostic implication of DPD quantification in ATTR-CA and made a significant contribution to the existing literature. Thirdly, the DPD retention index did not correlate with Perugini grades, likely due to the confounding effect of soft tissue uptake in planar imaging. However, our study cohort may not be representative of the entire ATTR-CA population. Fourthly, although the DPD retention index demonstrated significant associations with echocardiographic, laboratory, and clinical characteristics, the reported correlations were weak to modest. Fifthly, the results are based on DPD imaging and therefore may not be applicable to PYP imaging. In addition, there may be deviations between the DPD retention index and CPA or CPV when correlating with disease burden and prognostic factors in ATTR-CA. Therefore, further imaging studies are warranted to investigate the relationship between DPD and PYP quantification and their impact on outcome.

## Conclusion

In ATTR-CA, enhanced quantitative LV DPD uptake indicates advanced disease severity and is associated with adverse outcome. Therefore, DPD quantification may facilitate prognostic stratification when diagnosing patients with ATTR-CA.

## Supplementary data

Supplementary data are available at *European Heart Journal - Cardiovascular Imaging* online.

## Supplementary Material

jeae295_Supplementary_Data

## Data Availability

The data supporting the findings of this study are available from the corresponding author upon reasonable request.
