# Supplementary material for: Prognostic implication of DPD quantification in transthyretin cardiac amyloidosis
Source: Eur Heart J Cardiovasc Imaging. 2024 Nov 15;26(2):251–60. doi: 10.1093/ehjci/jeae295 (PMC11781830; doi:10.1093/ehjci/jeae295)
Supplement: jeae295_Supplementary_Data [file jeae295_supplementary_data.zip › Supplementary Table S1.docx]

**Supplementary Table S1.** Baseline characteristics and cohort comparison according to Perugini classification.

|  | All Patients  (n=100) | Perugini grade 2  (n=28) | Perugini grade 3  (n=72) | p |
| --- | --- | --- | --- | --- |
| Demographics |  |  |  |  |
| Age, years | 79.0 (75.0-84.0) | 79.5 (76.0-83.5) | 79.0 (74.0-84.0) | 0.656 |
| Male sex | 79 (79.0) | 23 (82.1) | 56 (77.8) | 0.630 |
| Body mass index, kg/m² | 25.7 (22.6-28.4) | 25.9 (23.0-28.3) | 25.7 (22.6-28.4) | 0.584 |
| ATTR variant | 13 (13.0) | 0 (0.0) | 13 (18.1) | **0.017** |
| Comorbidities |  |  |  |  |
| Atrial fibrillation/flutter | 62 (62.0) | 18 (64.3) | 44 (61.1) | 0.769 |
| Cardiac device | 23 (23.0) | 7 (25.0) | 16 (22.2) | 0.767 |
| Polyneuropathy | 61 (61.0) | 11 (39.3) | 50 (69.4) | **0.005** |
| Carpal tunnel syndrome | 48 (48.0) | 9 (32.1) | 39 (54.2) | **0.048** |
| Medications |  |  |  |  |
| Beta-blockers | 45 (45.0) | 15 (53.6) | 30 (41.7) | 0.283 |
| Diuretic agent | 76 (76.0) | 20 (71.4) | 56 (77.8) | 0.504 |
| Mineralocorticoid receptor antagonist | 53 (53.0) | 14 (50.0) | 39 (54.2) | 0.708 |
| Clinical Characteristics |  |  |  |  |
| NYHA functional class ≥ III | 46 (46.0) | 12 (42.9) | 34 (47.2) | 0.694 |
| NAC stage |  |  |  |  |
| I | 52 (52.0) | 17 (60.7) | 35 (48.6) | 0.277 |
| II | 32 (32.0) | 6 (21.4) | 26 (36.1) | 0.158 |
| III | 16 (16.0) | 5 (17.9) | 11 (15.3) | 0.752 |
| 6-minute walk distance, m | 363.0 (260.5-463.0) | 350.0 (264.0-496.0) | 365.0 (257.0-463.0) | 0.765 |
| Laboratory Characteristics |  |  |  |  |
| NT-proBNP, ng/L | 2341 (1248-3921) | 1960 (673-3134) | 2492 (1365-4008) | 0.141 |
| Troponin T, ng/L | 52.0 (32.0-72.0) | 41.5 (28.5-70.5) | 56.0 (40.0-77.0) | 0.116 |
| eGFR, mL/min/1.73m^2^ | 59.3 (44.0-78.7) | 70.0 (44.0-84.2) | 57.3 (43.8-73.4) | 0.131 |
| Nuclear Imaging Characteristics |  |  |  |  |
| Perugini grade 2 | 28 (28.0) | 28 (28.0) | 0 (0.0) | **<0.001** |
| Perugini grade 3 | 72 (72.0) | 0 (0.0) | 72 (72.0) | **<0.001** |
| DPD retention index, g/mL | 5.4 (3.4-7.9) | 4.6 (2.6-6.7) | 5.7 (3.7-8.5) | 0.086 |
| DPD activity, MBq | 720.0 (707.0-739.0) | 725.5 (710.5-732.0) | 716.0 (706.0-740.0) | 0.858 |
| DLP, mGy*cm | 85.0 (72.9-117.0) | 91.0 (79.0-119.0) | 81.0 (64.0-117.0) | 0.249 |
| Echocardiographic Characteristics |  |  |  |  |
| Interventricular septum, mm | 19.0 (16.0-22.0) | 18.0 (16.0-20.0) | 19.0 (17.0-23.0) | 0.209 |
| LV end-diastolic diameter, mm | 41.5 (37.0-46.0) | 43.0 (39.0-48.0) | 41.0 (37.0-44.0) | 0.201 |
| LV ejection fraction, % | 49.0 (40.0-55.0) | 51.0 (45.0-56.0) | 46.0 (39.7-54.8) | 0.344 |
| LV global longitudinal strain, -% | 11.0 (14.1-8.7) | 12.4 (14.4-10.0) | 10.8 (14.1-8.4) | 0.196 |
| E/e’ septal^*^ | 20.8 (16.8-25.5) | 18.8 (15.6-22.3) | 21.4 (17.0-26.4) | 0.135 |
| E/e’ lateral^*^ | 15.3 (12.4-20.2) | 12.8 (12.1-17.8) | 16.2 (13.2-21.6) | 0.115 |
| E/e’ average^*^ | 17.8 (14.7-23.4) | 16.2 (13.8-20.0) | 18.3 (15.2-24.2) | 0.098 |
| RV end-diastolic diameter, mm | 33.0 (30.0-37.0) | 32.0 (29.0-36.0) | 34.0 (30.0-37.0) | 0.298 |
| RV free wall longitudinal strain, -% | 14.1 (17.3-9.0) | 12.7 (17.0-9.0) | 14.7 (19.0-9.0) | 0.552 |
| LA volume index, mL/m2 | 35.1 (29.1-48.3) | 33.6 (28.9-54.3) | 35.9 (29.4-47.0) | 0.929 |
| RA volume index, mL/m2 | 32.8 (25.2-40.1) | 31.7 (25.4-38.7) | 33.7 (24.9-41.1) | 0.616 |
| TR velocity, m/s | 3.0 (2.6-3.3) | 3.0 (2.6-3.3) | 3.0 (2.7-3.3) | 0.813 |
| sPAP, mmHg | 48.0 (41.0-53.0) | 48.0 (37.0-54.0) | 48.0 (41.0-53.0) | 0.873 |

Continuous variables are expressed as median and interquartile range and categoric variables as numbers and percentages.

Bold indicates *p* ≤ 0.05. **^*^** E/e′ available for n=66 (Perugini grade 2: n=17, Perugini grade 3: n=49).

Abbreviations as in *Table 1.*
