# Supplementary figures and images for: Prognostic implication of DPD quantification in transthyretin cardiac amyloidosis
Source: Eur Heart J Cardiovasc Imaging. 2024 Nov 15;26(2):251–60. doi: 10.1093/ehjci/jeae295 (PMC11781830; doi:10.1093/ehjci/jeae295)

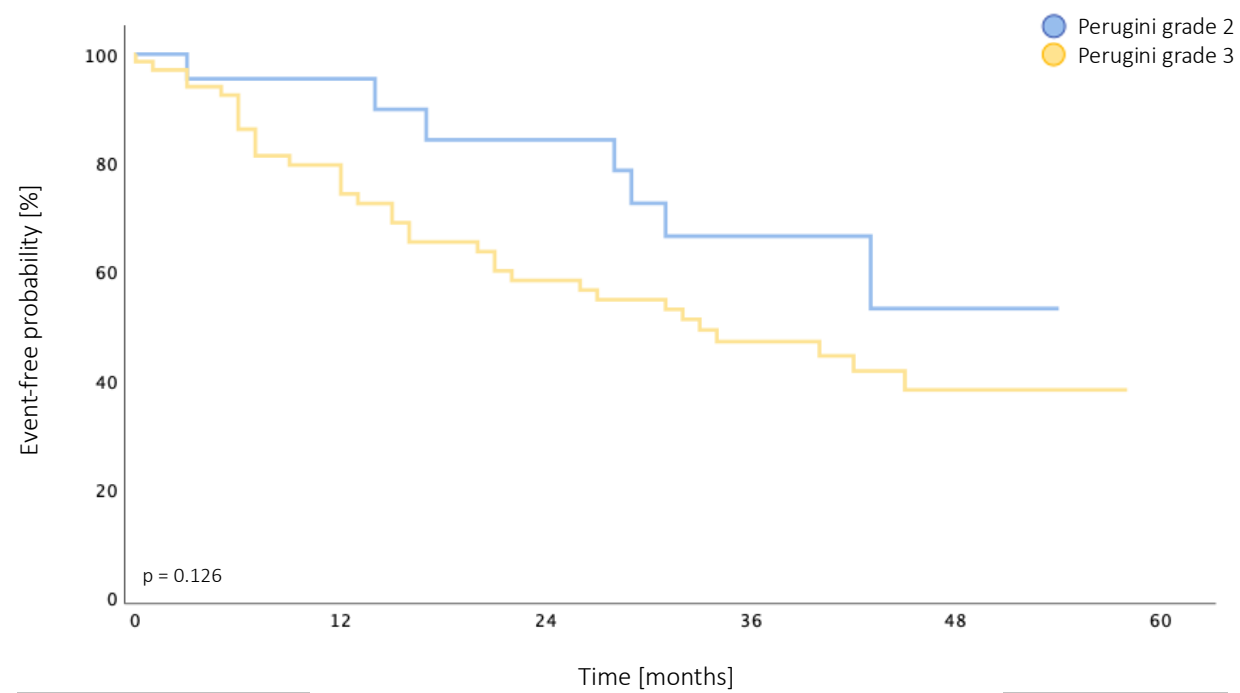

Supplement: jeae295_Supplementary_Data [file jeae295_supplementary_data.zip › Supplementary Figure S1.pdf]
